# Anatomical reduction and fixation of reverse Hill–Sachs lesion: description of a surgical technique

**DOI:** 10.1007/s00590-025-04499-8

**Published:** 2025-09-01

**Authors:** Michele Reboli, Francesco Bosco, Ilaria Zorzolo, Raul Alonso

**Affiliations:** 1 Department of Orthopaedics and Traumatology, Maria Vittoria Hospital, Turin, Italy, Turin, Italy; 2 Department of Precision Medicine in Medical, Surgical and Critical Care (Me.Pre.C.C.), University of Palermo, Palermo, Italy, Palermo, Italy; 3 Department of Orthopaedics and Traumatology, G.F. Ingrassia Hospital Unit, Palermo, Italy, Palermo, Italy; 4 Shoulder and Elbow Surgery Group, Cuf Tejo Hospital, Lisbon, Portugal, Lisbon, Portugal

**Keywords:** Reverse Hill–Sachs, Posterior shoulder dislocation, Shoulder instability, Anatomical reduction, Joint congruency

## Abstract

Reverse Hill–Sachs lesions pose a complex challenge in the acute management of posterior shoulder dislocations, often resulting in joint incongruity and compromised function. This article describes a surgical technique for the anatomical reduction and fixation of humeral head impaction injuries, aiming to restore the native sphericity of the articular surface and optimize joint stability. The proposed technique enables the precise realignment of the humeral cartilage and subchondral bone, thereby minimizing residual deformity and preserving the biomechanical integrity of the glenohumeral joint. When indicated, this approach offers a valuable alternative to traditional reconstructive procedures, potentially improving functional outcomes by preventing chronic instability and degenerative changes. The rationale, surgical steps, and key technical considerations are outlined, highlighting the advantages of early intervention in mitigating long-term sequelae.

**Supplementary Information:**

The online version contains supplementary material available at 10.1007/s00590-025-04499-8.

## Introduction

The reverse Hill–Sachs lesion (rHSl) is an impaction injury affecting the anteromedial surface of the humeral head, occurring in up to 90% of posterior shoulder dislocations [1]. When this lesion involves a significant portion of the humeral head—typically estimated at around 20% of its articular surface based on axial computed tomography scans—surgical intervention is indicated to achieve the following objectives: prevent recurrent glenohumeral dislocations by reducing engagement of the rHSl with the posterior glenoid rim, ensure a pain-free range of motion (ROM), optimize functional recovery, and minimize the risk of secondary glenohumeral osteoarthritis. In cases where the osteochondral defect is too extensive to allow joint-preserving procedures, shoulder replacement remains the only viable solution. The literature has extensively described the diagnostic and therapeutic characteristics of rHSl [1, 2]. However, comparative evidence between anatomical and non-anatomical techniques remains scarce, with no recent clinical studies beyond those already cited. The systematic review by van der List et al. in 2024 [1] identified key gaps, including incomplete and non-standardized ROM reporting, with internal rotation being reported in only 8/26 studies, and short follow-up. Our technique is based on the rationale that restoring the entire angular segment of the humeral head should optimize ROM, particularly internal rotation, warranting future comparative studies.

Currently, most surgically treated rHSl cases are managed using non-anatomical techniques, such as subscapularis tenodesis or lesser tuberosity transposition within the lesion. These approaches have demonstrated excellent results in preventing recurrent instability [1]. However, their impact on shoulder motion—particularly internal rotation in a semi-abducted position—remains unclear and is only anecdotally reported in the literature [3, 4]. The osteochondral defect reduces the available articular surface for glenohumeral articulation, which can be schematized as an angular segment of a semicircle in axial CT imaging. It is reasonable to assume that permanently altering this angular segment may lead to variable restriction of internal rotation using non-anatomical techniques.

Anatomical reduction and fixation of rHSl focus on restoring the continuity of the humeral articular cartilage and maintaining joint congruity by reinstating the lost angular section following trauma. Various surgical strategies have been discussed in the literature, including autologous anatomical reduction performed via open techniques [5] or arthroscopic methods [6, 7]. Options for addressing the defects also encompass reduction techniques that may utilize bone grafting or synthetic substitutes [6, 8, 9], as well as the application of osteochondral allografts to fill the humeral defect [4]. Autologous osteochondral grafting from the contralateral humerus has also been proposed in select cases of bilateral rHSl requiring prosthetic replacement on one side [10].

This study aims to describe a surgical technique for the anatomical reduction and fixation of rHSl through an open approach. By restoring the native humeral head morphology and preserving articular congruity, this technique offers a joint-preserving alternative that may optimize functional recovery and minimize the long-term risk of degenerative changes.

Video 1 illustrates the step-by-step execution of the surgical technique, providing a demonstration of the key procedural steps and technical details.

### Indications

The ideal candidate for this surgical technique is a patient with an acute rHSl, diagnosed within the first two weeks post-trauma, involving a substantial portion of the humeral articular surface (20–50%) and associated with a high risk of recurrent instability and/or significant restriction of joint mobility. Surgical intervention is contraindicated when the impaction lesion exceeds 50% of the articular surface or when the lesion is chronic. Relative contraindications include poor bone quality, preexisting osteoarthritic degeneration, delayed treatment beyond two weeks, and lesions where cartilage quality is presumed or confirmed to be degenerative. Although a standardized decision-making algorithm cannot be defined from the current literature [1, 2], we consider this technique feasible in the presence of early-stage chondromalacia, provided that the extent of the rHSl, the degree of chondral damage, and the patient’s age are carefully evaluated. Intraoperative conversion to arthroplasty is not regarded as a routine option, as its indications are distinct. When criteria for joint preservation are met, anatomical reconstruction may restore humeral head sphericity, thereby improving stability and ROM.

### Operating room setting

The procedure is performed with the patient positioned in a semi-sitting beach chair position under general anesthesia, with the possible addition of a peripheral nerve block for postoperative analgesia. A double aseptic surgical field is prepared, including both the operative shoulder and the contralateral iliac crest, in case bone grafting is required. The fluoroscopic device is positioned contralaterally or at the head of the patient to ensure optimal intraoperative visualization.

### Surgical approach and exposure of the impact lesion

A standard deltopectoral approach is utilized without the need for distal extension. After incising the deltopectoral fascia, the cephalic vein is identified, carefully protected, and retracted laterally. The deltopectoral interval is then developed to determine the coracoid process and the conjoint tendon, which are essential anatomical landmarks for the incision of the clavipectoral fascia. Utilizing the conjoint tendon as a medial reference, the clavipectoral fascia is incised and opened vertically, facilitating sufficient mobilization of the deltoid while maintaining the integrity of the subdeltoid bursa. This careful approach ensures that surrounding structures are preserved, optimizing the surgical field for subsequent procedures. The lesser tuberosity of the humerus, along with the insertion of the subscapularis tendon and the long head of the biceps tendon, is subsequently identified. The biceps tendon is isolated and used as a guide to access the rotator interval.

**Fig. 1:**
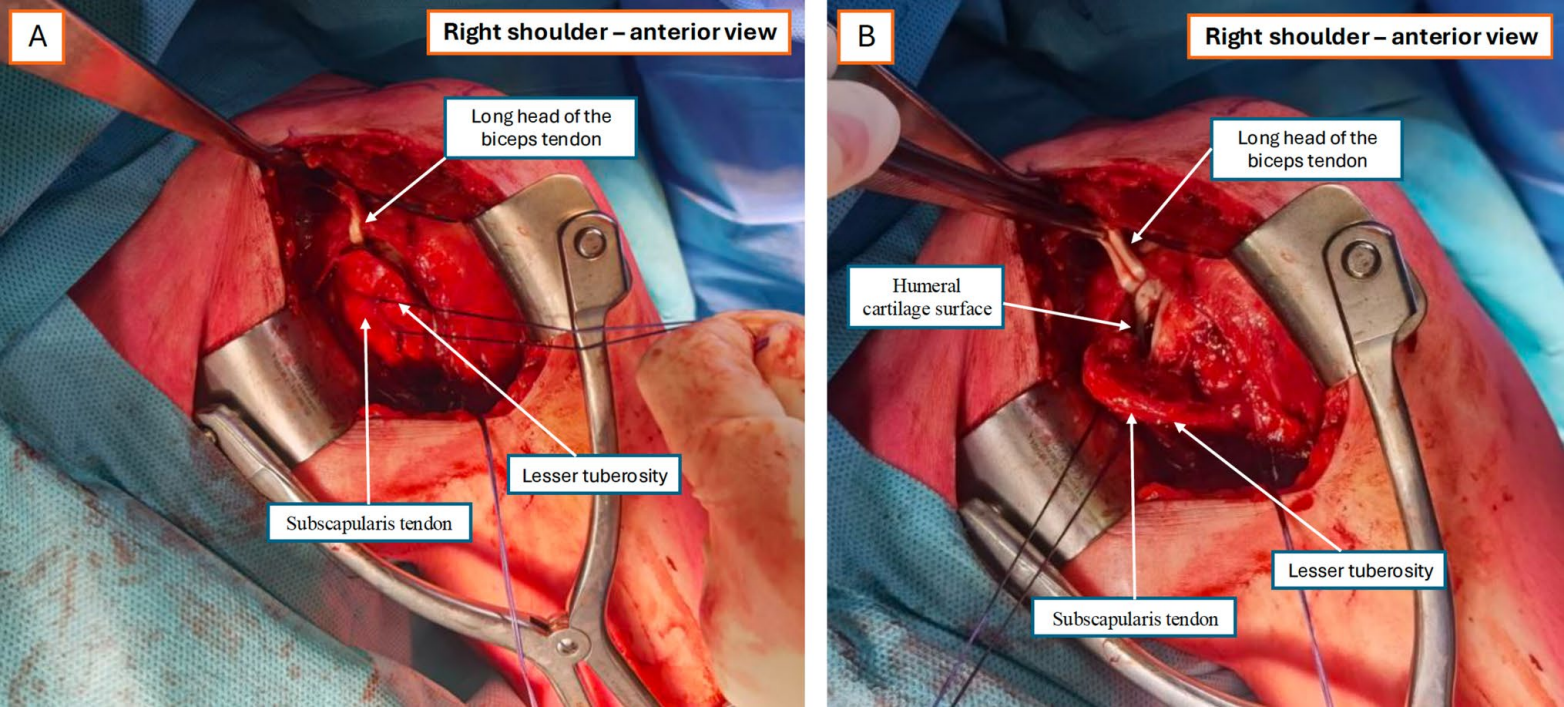
Exposure through the deltopectoral approach and osteotomy of the lesser tuberosity. **A** Identification of the long head of the biceps tendon, subscapularis tendon, and lesser tuberosity; **B** Osteotomy line of the lesser tuberosity exposing the humeral cartilage surface and reverse Hill–Sachs lesion area. Orientation: right shoulder—anterior view

At this stage, a tenodesis of the long head of the biceps to the pectoralis major tendon is performed. The lesser tuberosity is then skeletonized, and the subscapularis tendon is reinforced with two sutures (Fig. 1A). An osteotomy of the lesser tuberosity is executed using a 10 mm osteotome, progressing in a lateral-to-medial direction to create a bone flap that allows direct access to the humeral cartilage surface and the rHSl (Fig. 1B). During the medial mobilization of the osteotendinous flap formed by the lesser tuberosity and the subscapularis tendon, it is crucial to avoid excessive tension on the lower third of the flap to preserve vascularization, which is primarily supplied by the anterior circumflex vessels.

### Reduction and stabilization of the impact lesion

After exposing the rHSl osteochondral impaction lesion, its extent and characteristics are carefully assessed to formulate an optimal reduction strategy. When feasible, a controlled incomplete osteotomy is performed on the anterolateral humeral head, preserving the intact cartilaginous surface as a hinge to allow elevation and reduction of the impacted osteochondral segment. This technique maintains a stable fulcrum, enabling restoration of humeral head sphericity and articular congruity. If preservation of the hinge is not possible, complete osteochondral mobilization is performed according to the clinical scenario.

**Fig. 2:**
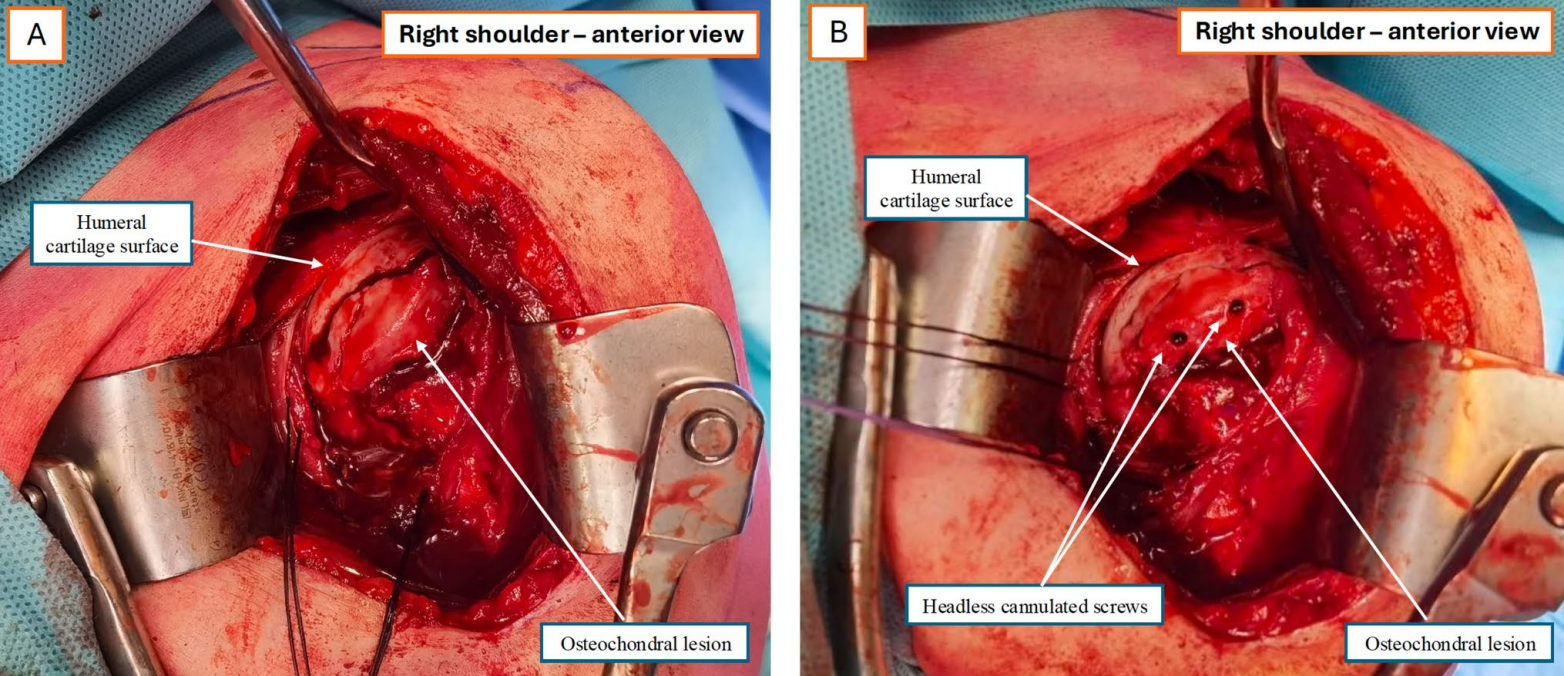
Reduction and fixation of the impacted osteochondral fragment. **A** Incomplete osteotomy on the anterolateral humeral head preserving a chondral hinge; humeral cartilage surface and osteochondral lesion visible. **B** Final fixation with headless cannulated screws; restored humeral head sphericity. Orientation: right shoulder—anterior view

Once reduction is achieved (Fig. 2A), the fragment is fixed with headless cannulated screws (Fig. 2B). Fluoroscopy is routinely performed at the end of fixation to verify screw length, exclude intra-articular placement, and reassess tuberosity repair. Given the wide exposure obtained, intraoperative fluoroscopy after guidewire placement is generally unnecessary.

The stability of the anatomical reconstruction is then assessed through a full ROM to ensure adequate fixation and joint congruity.

### Closure of the lesser tuberosity osteotomy

The osteotomized lesser tuberosity of the humerus is anatomically reduced and stabilized using two 5 mm titanium anchors, each with two strands, positioned at the medial end of the osteotomy. The strands are passed through the subscapularis tendon at the tendon–bone interface (pre-insertional region), and the medial strand of each pair is secured with five simple knots to ensure robust fixation.

**Fig. 3:**
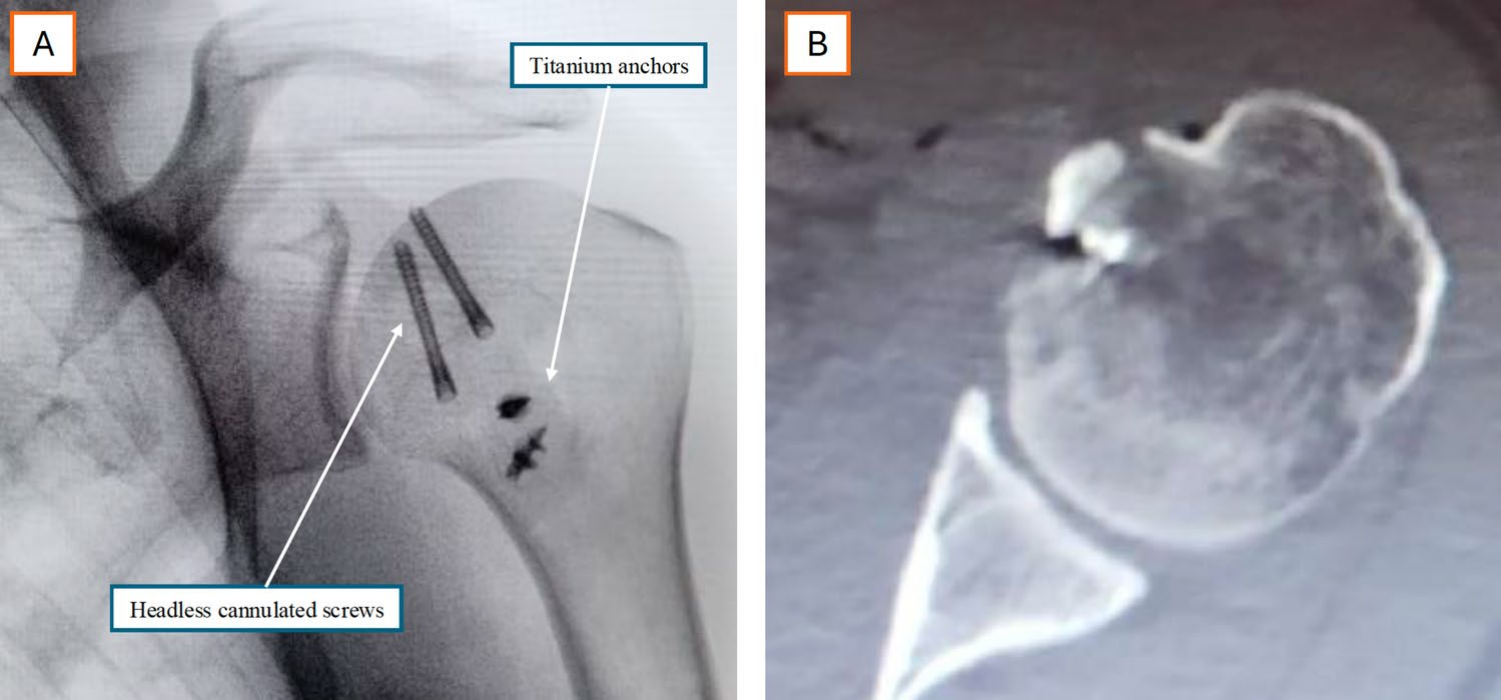
Postoperative imaging. **A** Anteroposterior radiograph showing headless cannulated screws and titanium anchors for tuberosity fixation. **B** Axial CT scan confirming restoration of the articular surface. Orientation: right shoulder

To further reinforce the construct, three distinct bone tunnels are created at the lateral margin of the osteotomy, emerging at the bicipital groove at approximately 5 mm intervals. The tails of the anchor strands are passed, using a shuttle and a transport wire, through these tunnels—distributing two strands through the upper and lower holes and four strands through the central hole—before being secured with simple knots, completing the transosseous repair. The stability of the tuberosity fixation is then assessed through controlled internal and external rotation movements. Final fluoroscopic verification is performed, followed by thorough saline irrigation. The surgical wound is closed in layers, and the skin is sutured with an intradermal technique (Fig. 3). A flat dressing is applied, immobilizing the limb in a neutral rotation brace.

### Postoperative program

The patient's upper limb is maintained in a neutral position using a brace for five weeks postoperatively, both during daily activities and overnight. From the immediate postoperative period, passive flexion and extension movements of the elbow are permitted, and hand-opening and closing exercises are encouraged to prevent stiffness and maintain circulation.

At three weeks post-surgery, the patient begins targeted physiotherapy to gradually restore flexion, extension, and abduction mobility while maintaining a neutral rotational position. Controlled recovery of internal and external rotations is initiated at six weeks postoperatively. The patient is typically hospitalized overnight and discharged the following day with no complications.

Plain radiographs and clinical evaluations are scheduled at 30, 60, and 90 days postoperatively, followed by quarterly assessments to monitor clinical progress and ensure proper healing.

We do not follow standardized clinical or radiographic thresholds to advance rehabilitation; instead, the physiokinesitherapy program is adapted according to the biological timing considered safe, allowing for a gradual integration of exercises as outlined in the manuscript.

## Discussion

The main finding of this study is that open anatomical reduction and fixation of an rHSl is a viable surgical approach, enabling the restoration of the humeral head's native articular surface and potentially optimizing joint congruity and functional outcomes. This technique aims to prevent recurrent posterior instability and restore shoulder biomechanics by preserving the pre-traumatic anatomy as much as possible.

**Fig. 4:**
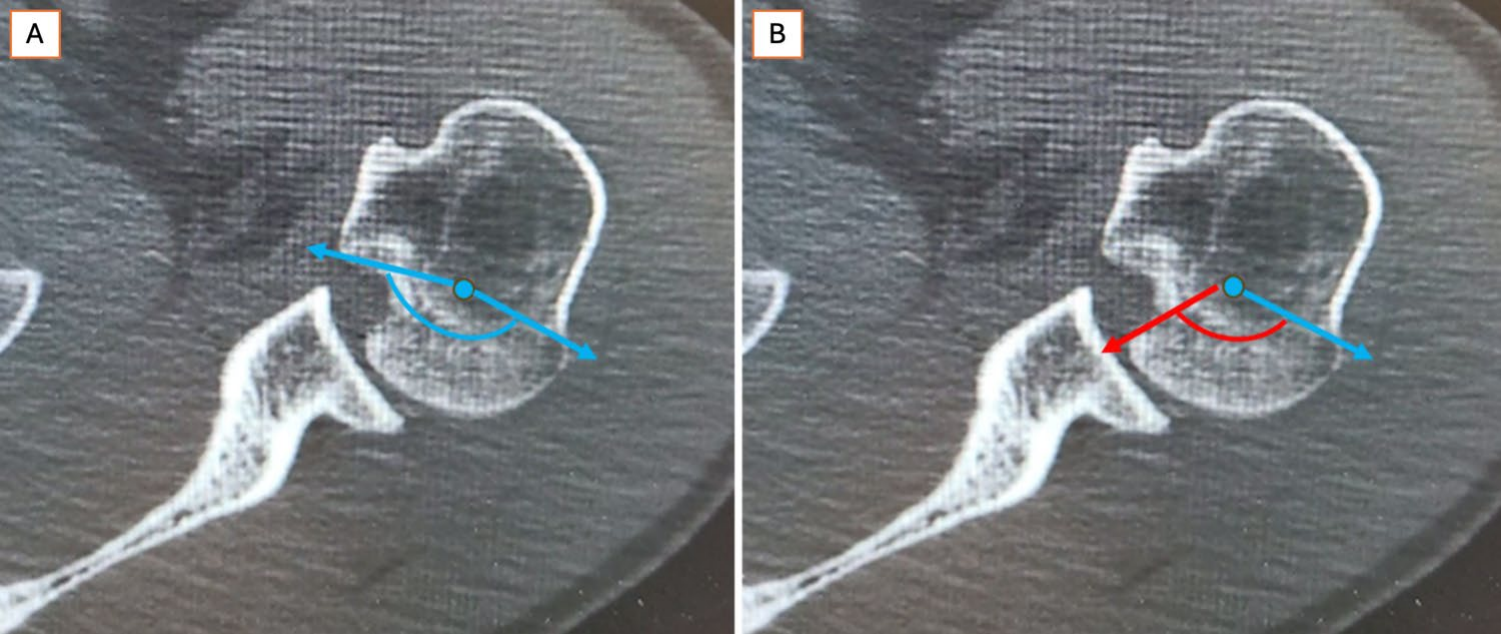
Schematic representation of the humeral head articular surface in axial CT view. (A) Axial CT schematic representation of the humeral head articular arc. **A** Pre-traumatic configuration: blue line with arrows indicating the intact articular arc, blue dot marking the humeral head center. **B** Post-traumatic condition showing the remaining articular arc (blue line) and deficit arc caused by the reverse Hill–Sachs lesion (red line), with posterior glenoid rim engagement zone indicated (red arrow), and blue dot marking the humeral head center. Orientation: right shoulder

It is essential to acknowledge that each clinical case presents unique anatomical variables, requiring an individualized surgical approach. As an anatomical reconstruction method, the proposed technique relies on early diagnosis and timely intervention to ensure that the osteochondral tissue is addressed in the best possible biological condition. Anatomical reduction of the impacted osteocartilaginous surface restores the lost angular section of the humeral head, which is crucial for maintaining smooth glenohumeral articulation. This reduction can be visualized in two dimensions on an axial CT slice as an angular section of a semicircle (Fig. 4).

Recent systematic reviews indicate that both anatomical and non-anatomical surgical techniques are effective in preventing further dislocation episodes [1–3]; however, there is a paucity of studies that thoroughly assess functional outcomes, particularly with a complete analysis of active (aROM) and passive range of motion (pROM) [1, 3]. Additionally, most studies have low methodological quality, a small sample size, lack long-term data, and often fail to provide crucial data on osteoarthritis progression, subscapularis healing, and ROM recovery—factors that are fundamental in determining the superiority of one technique over another [3]. Furthermore, literature on anatomical techniques is often inconsistent, as many studies do not specify the surgical method used, leading to confusion when results from different procedures are reported collectively [2, 3].

A notable confounding factor in the literature is the grouping of autologous anatomical reduction and fixation techniques together with the use of osteochondral allografts under the general category of "anatomical techniques" [3, 4]. We believe that distinguishing between these two approaches is essential for a more accurate assessment of clinical and radiographic outcomes. Autologous anatomical reduction techniques aim to restore the patient's native articular surface by disimpacting and fixating the humeral osteochondral fragment, potentially supported by a graft. In contrast, non-autologous reconstruction techniques rely on osteochondral allografts for defect management. Both methods aim to anatomically restore the sphericity of the humeral head after a posterior shoulder dislocation with associated rHSl. The use of osteochondral allografts, although making it possible to treat even larger and chronic impact lesions, is subject to risks such as graft resorption, potential infectious dangers, and outcomes that may be less favorable compared to those of an autologous reconstruction technique.

Several authors have attempted to restore the pre-traumatic anatomy of rHSl using various approaches, including both open and arthroscopic methods, with or without graft augmentation [4–8]. Regardless of the specific technique, we consider restoring the humeral articular surface and joint congruity as mandatory objectives for the orthopedic surgeon. Achieving these goals is critical not only for preventing recurrent instability but also for optimizing long-term functional outcomes.

### Strengths and limitations

The primary strength of this technique lies in its ability to restore the native sphericity of the humeral head, thereby preserving joint congruity and optimizing biomechanical function. Unlike non-anatomical procedures, which may alter shoulder kinematics, this approach aims to maintain the pre-traumatic anatomy, potentially reducing the risk of motion restrictions and secondary osteoarthritis. Additionally, early intervention enables the utilization of osteochondral tissue in its optimal biological condition, which may enhance healing potential and improve long-term outcomes. In contrast to previously described techniques that employ structural grafts, such as osteochondral allografts, our method achieves articular surface restoration exclusively using the native osteochondral fragment, thereby avoiding graft-related complications, including immune reactions, resorption, or disease transmission. Several alternative techniques have been described for the management of reverse Hill–Sachs lesions, including arthroscopic-assisted reduction and fixation, tendon transfer procedures such as the modified McLaughlin, and autologous bone graft reconstruction [11–13]. While these approaches may achieve satisfactory outcomes in selected cases, they differ substantially from our technique, which restores the native articular surface exclusively using the original osteochondral fragment, thereby avoiding the potential risks associated with graft harvesting or incorporation.

However, this technique also presents some limitations. It requires a timely diagnosis and intervention within two weeks post-trauma, which may not always be feasible in clinical practice. The procedure is technically demanding, requiring precise osteotomy execution and stable fixation to achieve satisfactory results. Moreover, patient selection is crucial, as poor bone quality, extensive cartilage degeneration, or delayed treatment may compromise the effectiveness of the anatomical reconstruction. The relatively small sample size of the present study further limits the generalizability of our findings. Additionally, this report is based on a single surgical technique without associated clinical outcome data; to address this evidence gap, a prospective case series with standardized functional and radiographic follow-up is currently being designed to evaluate mid- and long-term results. Lastly, while this approach aims to restore the native joint surface, long-term studies assessing functional outcomes, residual pain, and osteoarthritis progression are still limited, underscoring the need for further research to establish its long-term efficacy.

## Conclusion

Anatomical reduction and fixation of rHSl should be considered a valid treatment option in the acute setting, provided that appropriate surgical indications are strictly followed. The technique presented offers the advantage of restoring the native morphology of the proximal humerus, thereby preserving joint congruity and biomechanics. We believe that the surgical insult associated with this approach is justified by the potential benefits of anatomical reconstruction, which not only helps prevent recurrent posterior instability but may also contribute to improved functional outcomes and long-term shoulder performance. Further studies with long-term follow-up are necessary to validate the effectiveness of this technique and compare its outcomes with those of alternative surgical approaches.

## Supplementary Information

Below is the link to the electronic supplementary material.

Supplementary file1 (MP4 141764 KB)

## Data Availability

No datasets were generated or analysed during the current study.
